# Isolated pancreatic metastasis from malignant melanoma: a case report and literature review

**DOI:** 10.1007/s12328-019-00996-6

**Published:** 2019-05-27

**Authors:** Yoshifumi Nakamura, Reiko Yamada, Maki Kaneko, Hiroaki Naota, Yu Fujimura, Masami Tabata, Kazuhiko Kobayashi, Kyosuke Tanaka

**Affiliations:** 1 Department of Gastroenterology, Matsusaka Chuo General Hospital, Matsusaka, Mie Japan; 2 Department of Gastroenterology and Hepatology, Mie University Hospital, 2-174 Edobashi, Tsu, Mie 514-8507 Japan; 3 Department of Endoscopy, Mie University Hospital, Tsu, Mie Japan; 4 Department of Surgery, Matsusaka Chuo General Hospital, Matsusaka, Mie Japan

**Keywords:** Malignant melanoma, Endoscopic ultrasound (EUS), Endoscopic retrograde cholangiopancreatogram (ERCP), Endoscopic ultrasound-guided fine-needle aspiration (EUS-FNA), Case report

## Abstract

Isolated pancreatic metastasis from malignant melanoma is rare. Pancreatic metastasis is difficult to diagnose in patients with unknown primary malignant melanoma. Endoscopic ultrasound-guided fine-needle aspiration plays an important role in confirming the diagnosis. A 67-year-old woman was referred to our institution because of a mass in her pancreas. Computed tomography and magnetic resonance imaging revealed a 35-mm mass localized on the pancreatic tail, with low attenuation, surrounded by a high-attenuation rim. Endoscopic ultrasonography revealed a hypoechoic mass with central anechoic areas. Endoscopic ultrasound-guided fine-needle aspiration of the mass was performed, and the pathological diagnosis was malignant melanoma. Intense fluorodeoxyglucose uptake was observed in the pancreatic tail on positron emission tomography–computed tomography. No other malignant melanoma was found. Distal pancreatectomy was performed. Six months postoperatively, positron emission tomography–computed tomography revealed high uptake in the left nasal cavity, and biopsy revealed the mass to be a malignant melanoma, indicating that the primary site of the malignant melanoma was the left nasal cavity and that the pancreatic mass and peritoneal lesion were metastases. The patient had survived > 2 years after the distal pancreatectomy. Pancreatic resection of isolated pancreatic metastasis can possibly prolong survival; however, metastatic melanoma usually has poor prognosis.

## Introduction

Pancreatic metastases are rare, ranging from 2 to 5% of pancreatic malignancies [1, 2]. The most common primary malignancies that metastasize to the pancreas are renal, lung, breast, and colon cancer, with sarcoma and melanoma observed less commonly [2–4]. Metastatic melanoma has a poor prognosis; the median survival for patients with stage IV melanoma ranges from 8 to 18 months after diagnosis [5]. Isolated pancreatic metastasis is a rare event that represents about less than 1% of metastatic melanomas [6].

Pancreatic metastases can resemble primary pancreatic malignancies, such as ductal carcinoma or neuroendocrine tumors. Thus, it can be difficult to differentiate pancreatic metastases from primary tumors based only on imaging findings. Endoscopic ultrasound-guided fine-needle aspiration (EUS-FNA) plays an important role in confirming the diagnosis [1]. There are only a few reports on surgically resected pancreatic metastasis of malignant melanoma diagnosed by EUS-FNA [3, 7, 8]. Here, we present a unique case of malignant melanoma with isolated pancreatic metastasis diagnosed by EUS-FNA and was treated with distal pancreatectomy.

## Case report

A 67-year-old woman, who had been healthy all her life, presented to the referring hospital with left upper quadrant abdominal pain. Her ultrasonogram and computed tomography (CT) showed a mass in the pancreas, and the patient was referred to our institution for further examination.

**Fig. 1:**
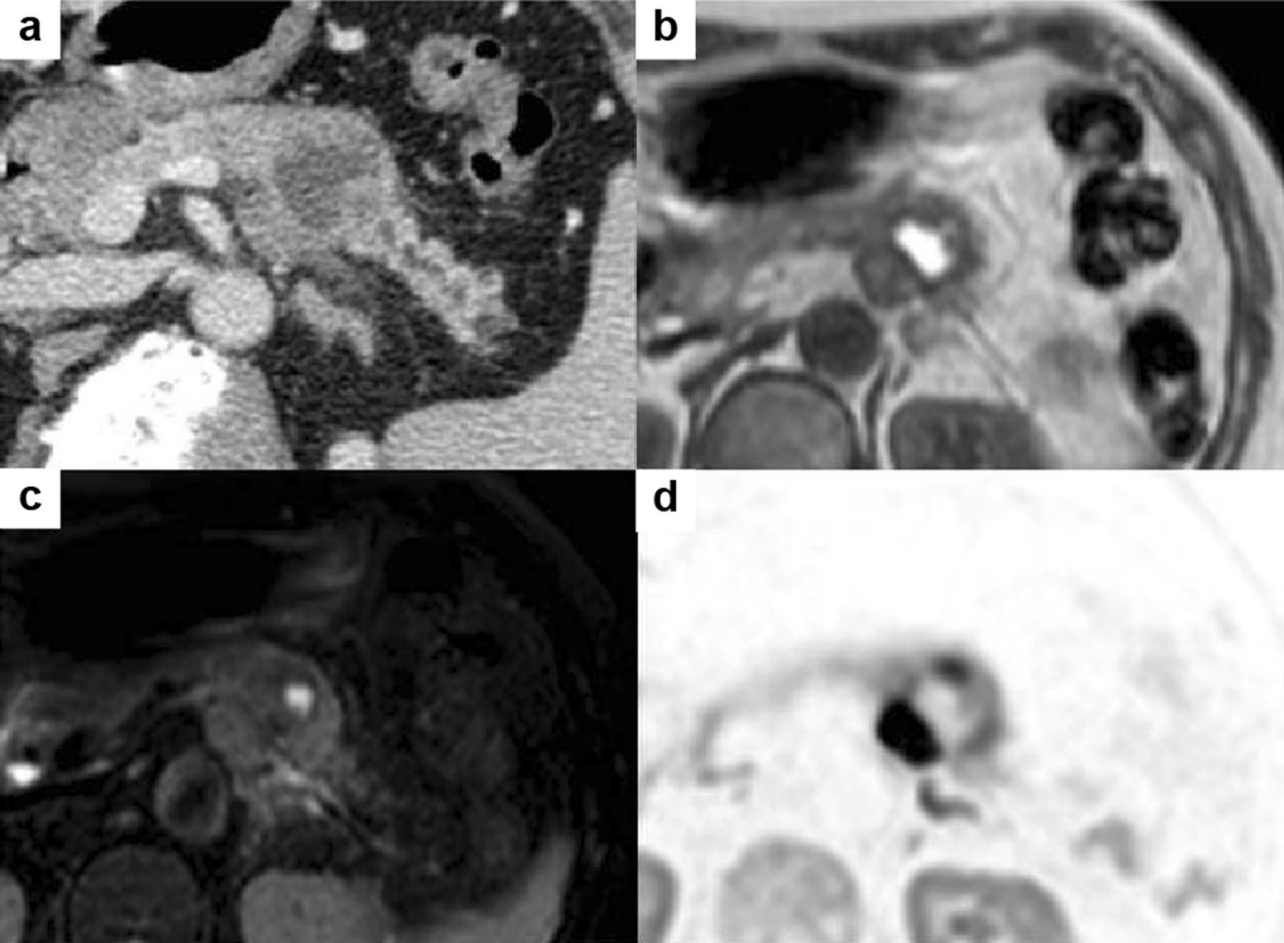
Computed tomography image. **a** Mass in the tail of the pancreas with pancreatic ductal dilation. The central mass is hyperintense on T1-weighted image (**b**) and hypointense on T2-weighted image (**c**). **d** Peripheral rim of the mass is hyperintense on diffusion-weighted image

**Fig. 2:**
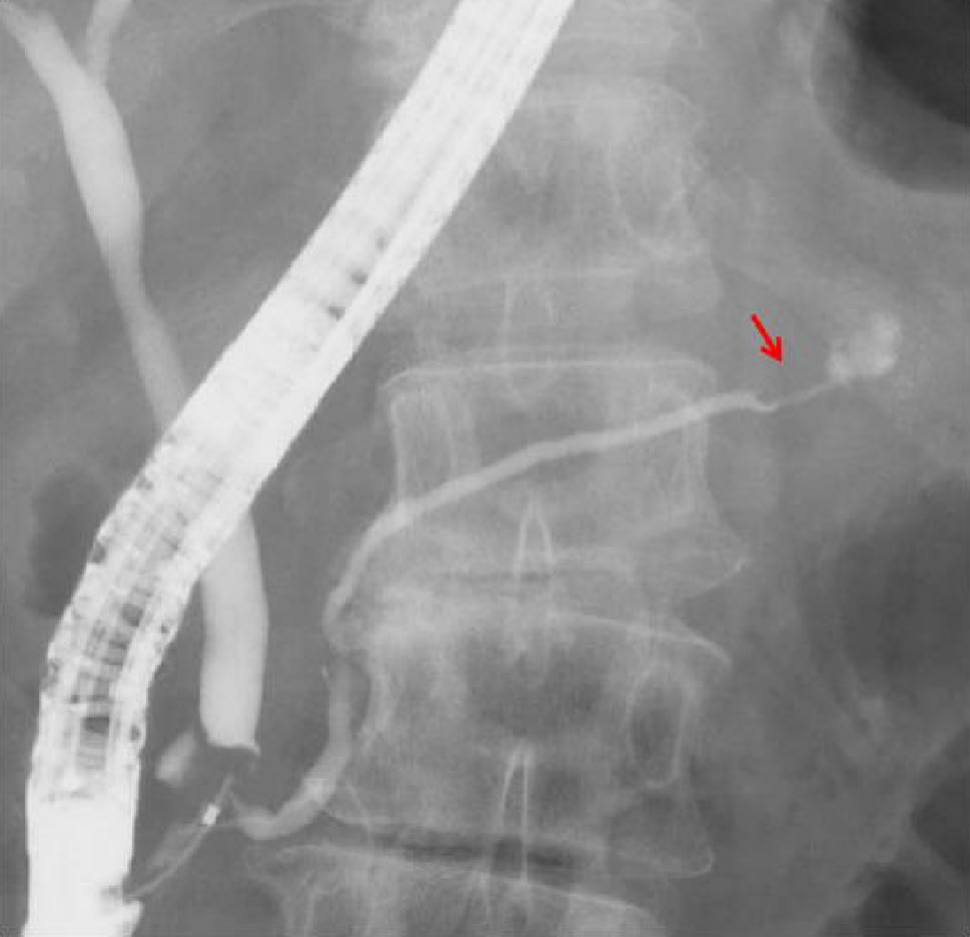
Endoscopic retrograde cholangiopancreatogram revealed smooth narrowing and displacement of the pancreatic duct with upstream dilatation

**Fig. 3:**
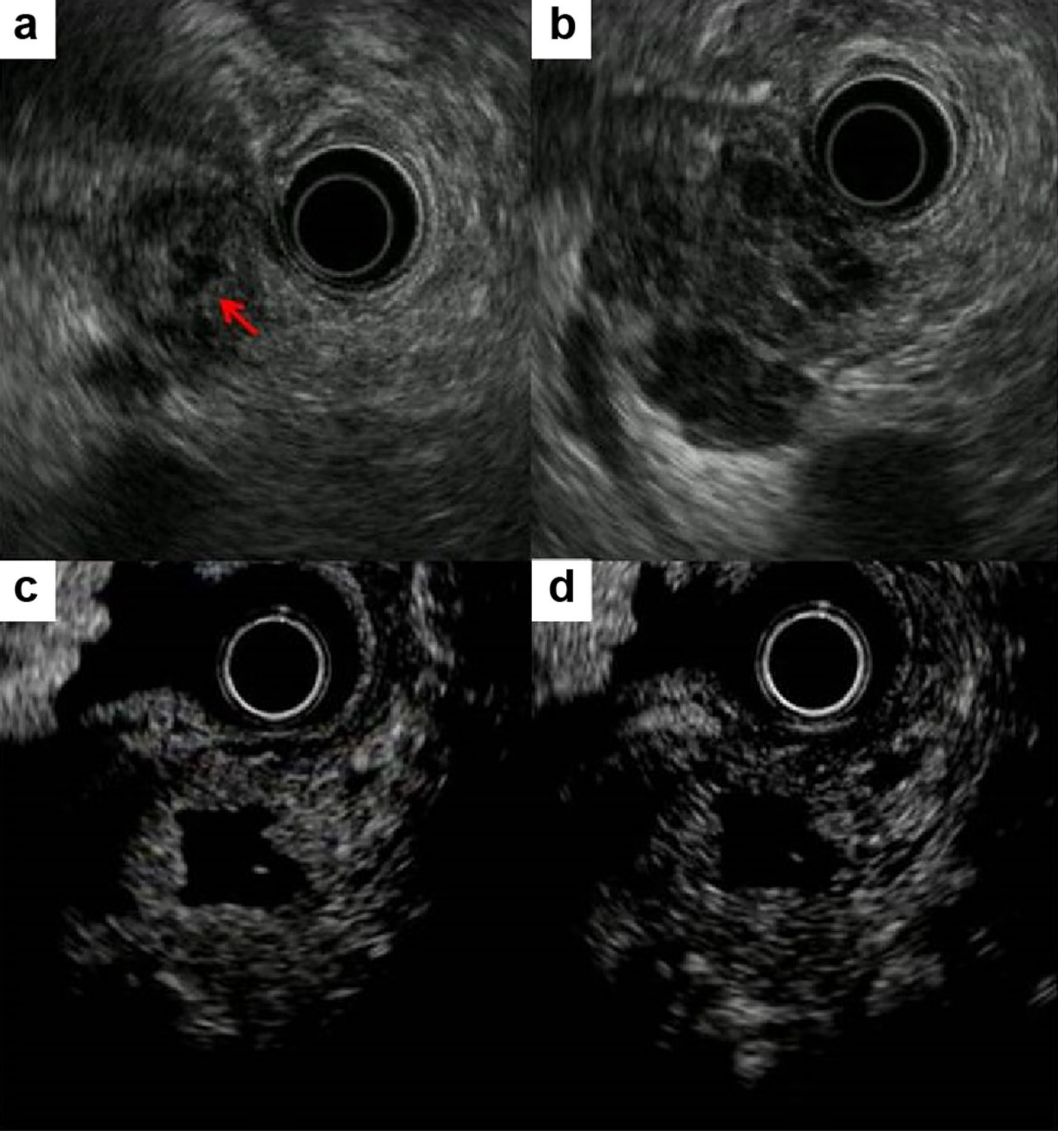
Endoscopic ultrasonography revealed hypoechoic and homogenous heterogeneous mass (**a**) with central anechoic areas (**b**, arrow). Contrast-enhanced endoscopic ultrasonography shows isoenhancement at 20 s (**c**) and hypoenhancement at 120 s (**d**) with central non-enhancement of the peripheral rim of the mass

Enhanced CT revealed that the mass was localized to the tail of the pancreas, with pancreatic ductal dilatation. The mass was a rounded, well-defined lesion with low attenuation, surrounded by a high-attenuation rim (Fig. 1a). Magnetic resonance imaging (MRI) showed that the center of the mass was hyperintense on T1-weighted image and hypointense on T2-weighted image (Fig. 1b, c). The diffusion-weighted image showed a hyperintense peripheral rim of the mass (Fig. 1d). Endoscopic retrograde cholangiopancreatogram demonstrated smooth narrowing and displacement of the pancreatic duct with upstream dilatation (Fig. 2). EUS revealed the 35-mm mass to be hypoechoic and heterogeneous with central anechoic areas (Fig. 3a, b). Contrast-enhanced EUS (CE-EUS) was conducted using an electronic radial-type endoscope (GF-UE260; Olympus, Japan) and perflubutane as ultrasound contrast agent. CE-EUS showed isoenhancement during the 20-s phase (Fig. 3c) and hypoenhancement during the 120-s phase (Fig. 3d) of the peripheral rim of the mass with central non-enhancement.

**Fig. 4:**
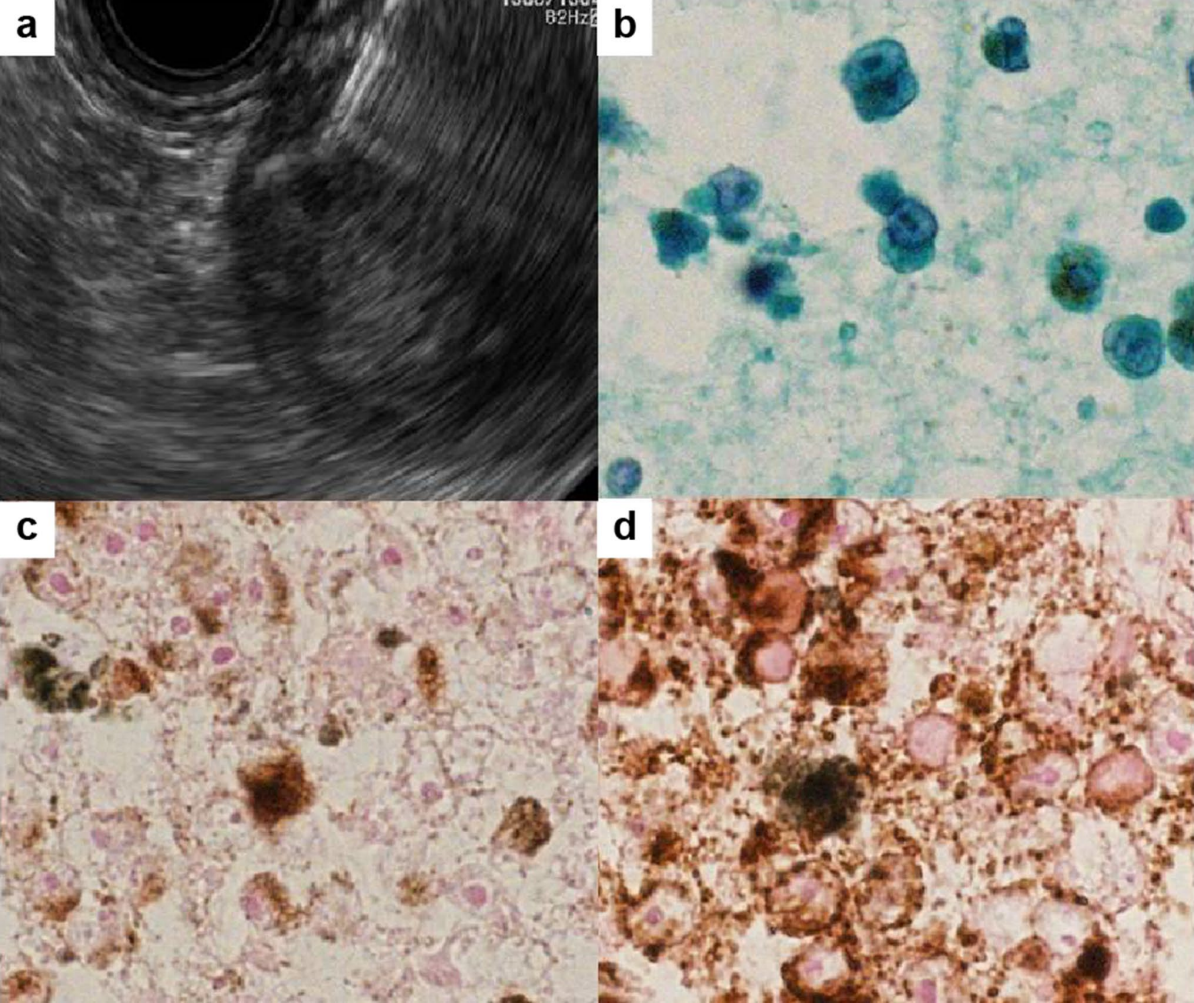
**a** Endoscopic ultrasound-guided fine-needle aspiration of the peripheral rim of the mass. **b** Cytologic results revealed a large nucleus and a high nuclear/cytoplasmic ratio in the cells, with brown pigmentation. Immunocytochemical staining with Melan A (**c**) and Human Melanoma Black 45 (**d**)

Cytological analysis obtained by EUS-FNA with a 22-gauge needle (Fig. 4a) revealed a large nucleus and a high nuclear/cytoplasmic ratio in the cells, with brown pigmentation (Fig. 4b). The cells were positive for Melan A and Human Melanoma Black 45 (HMB-45) and were negative for S100 on cell-block immunocytochemical analysis (Fig. 4c, d). Thus, the patient was diagnosed as having malignant melanoma.

**Fig. 5:**
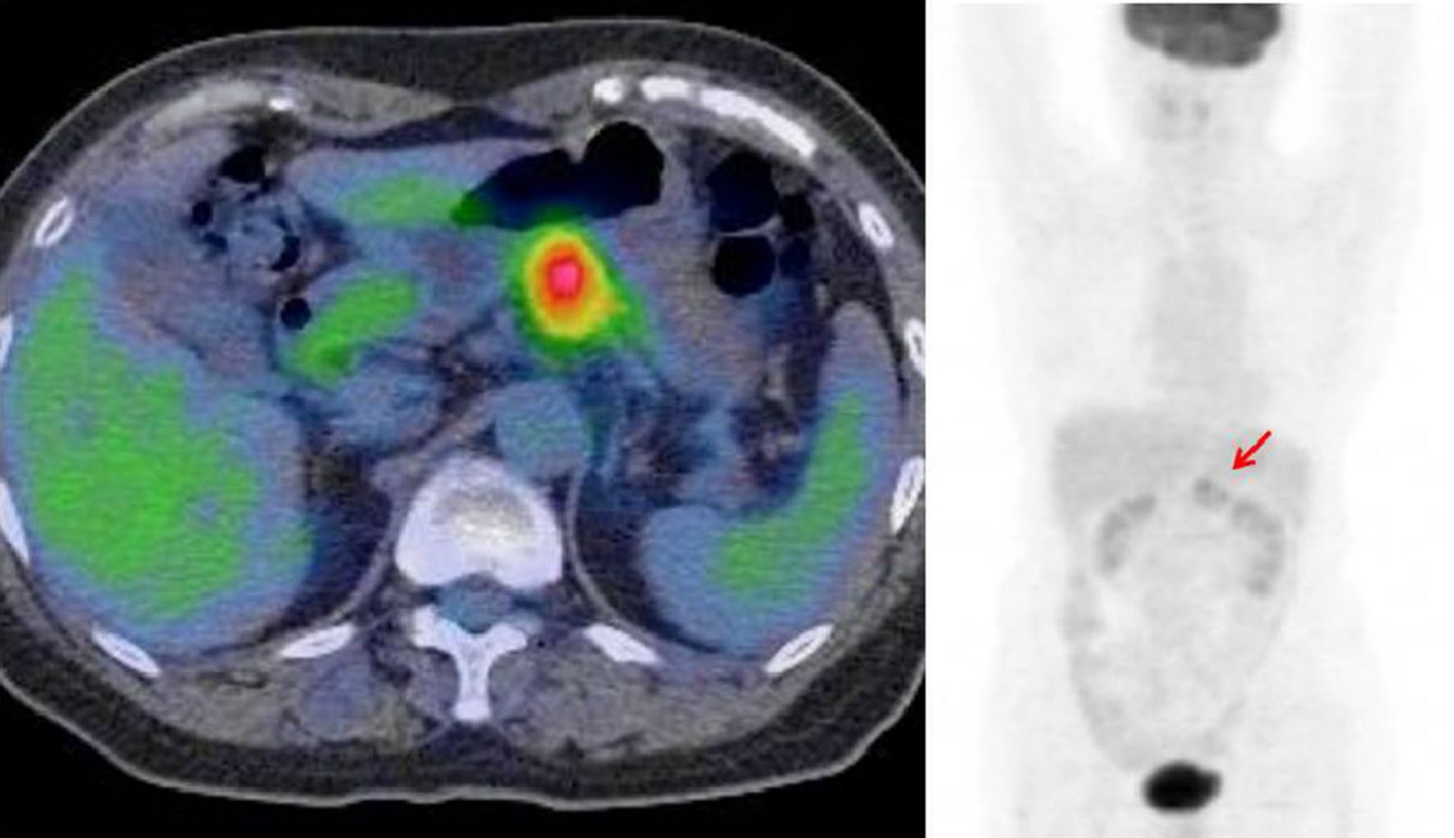
Intense fluorodeoxyglucose uptake only in the body and tail of the pancreas (arrow)

Since primary pancreatic malignant melanoma has never been reported before, we suspected metastatic malignant melanoma of the pancreas. However, intense fluorodeoxyglucose uptake was observed only in the tail of the pancreas on positron emission tomography–CT (PET-CT) (Fig. 5). Esophagogastroduodenoscopy and colonoscopy did not reveal any specific findings. The primary site could not be identified by dermatological, ophthalmological, or gynecological examination.

**Fig. 6:**
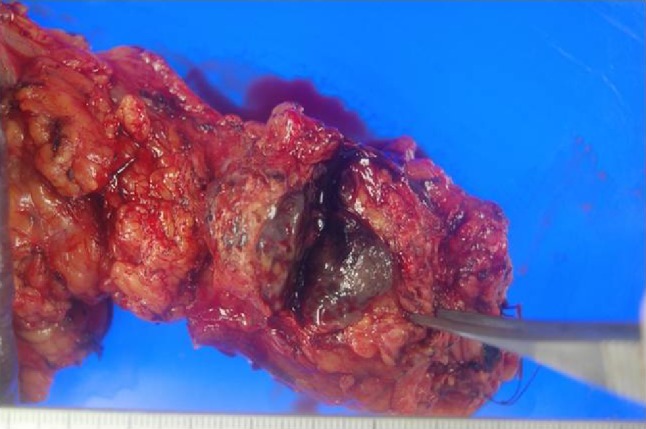
Resected surgical specimen showing a black–brown mass in the tail of the pancreas

**Fig. 7:**
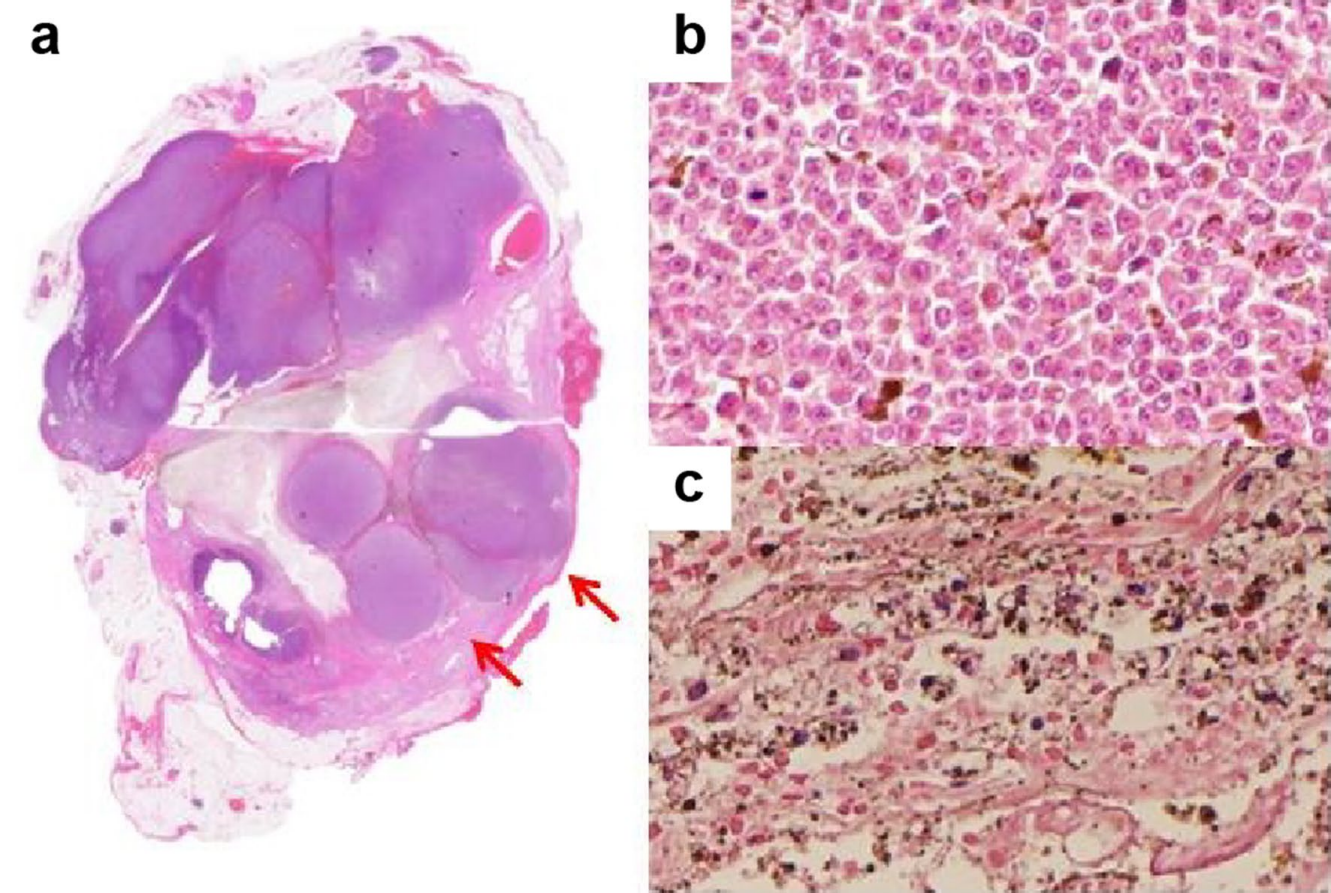
**a** Loupe image of the resection specimen. The peripheral rim of the mass has nodular components (arrows). **b** Tumor cells in the peripheral rim of the mass have anisokaryosis and clear nuclei with melanin production. **c** Center of the mass was necrotic

**Fig. 8:**
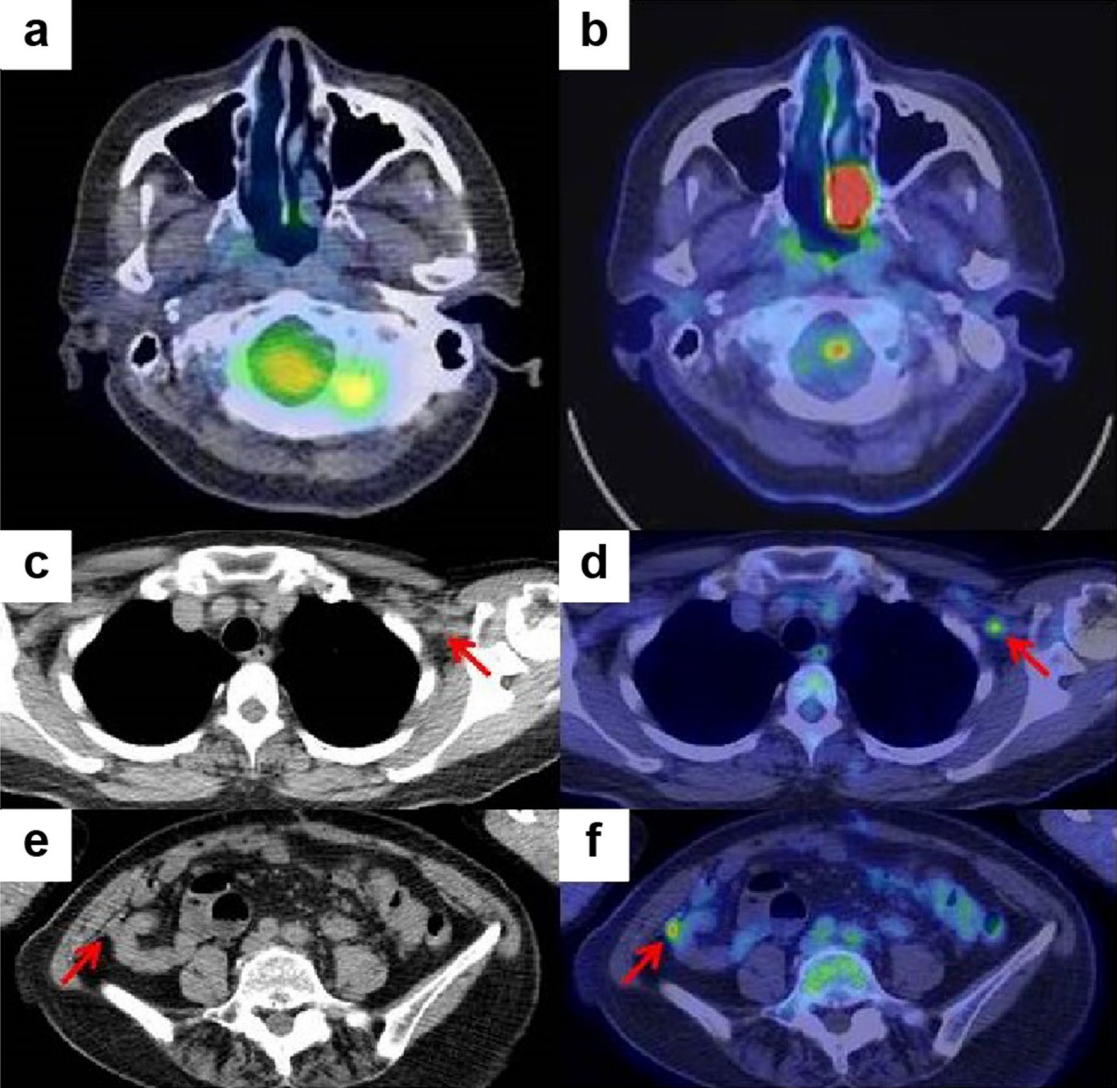
Positron emission tomography–computed tomography image of the nasal cavity before (**a**) and after surgery (**b**). Plane computed tomography and positron emission tomography–computed tomography images after surgery revealed left infraclavicular lymph node metastasis (**c**, **d**) and a small peritoneal nodule (**e**, **f**)

Distal pancreatectomy was performed. Histological examination of the surgical specimen revealed malignant melanoma with central necrosis (Figs. 6, 7). The resection specimen stained for Melan A and HMB-45, but not for S100. The patient underwent interferon-alfa treatment as an adjuvant therapy. Six months postoperatively, the follow-up PET-CT showed high uptake in the left nasal cavity, left infraclavicular lymph, and peritoneum (Fig. 8). On fiber-optic laryngoscopy, a whitish mass was detected in the left nasal cavity, which was determined to be a malignant melanoma. Although melanin was unclear in the nasal cavity biopsy specimen, cell shape and immunohistochemistry findings were the same as those in the resected surgical specimen. The primary site of the malignant melanoma was the left nasal cavity, and the pancreatic mass, left infraclavicular lymph, and peritoneal lesion were metastases. Nivolumab was started; thereafter, the treatment was switched to pembrolizumab. The patient had survived for more than 2 years after the distal pancreatectomy.

## Discussion

Malignant melanoma usually metastasizes to the gastrointestinal tract, and metastatic malignant melanoma usually affects multiple sites. Isolated organ metastasis is unusual; specifically, metastasis to the pancreas is extremely rare (< 1%) [6]. There are 76 cases of pancreatic metastasis from malignant melanoma reported in English (Table 1). The major primary site is cutaneous and ocular. Meanwhile, there are only three cases of pancreatic metastasis from nasal cavity malignant melanoma, including our case [9, 10]. Sometimes, the primary lesion of melanoma is difficult to identify during pretreatment evaluation. In our case, the primary site was identified by PET-CT 6 months postoperatively, even though PET-CT is only effective for detecting primary tumors or cancers of unknown primary.

**Table 1:** Metastatic malignant melanoma of pancreas reported in the English literature

Authors	Year of publication	Age	Sex	Case	Primary site	Location in the pancreas	Tumor size (cm)	Diagnostic modality	Surgery	Follow-up (month)	Outcome
Das Gupta et al. [21]	1964	44	Female	2	Cutaneous	Body and tail	NR	Exploratory laparotomy	No operation	2	Dead
		28	Male		Cutaneous	Body and tail	NR	Exploratory laparotomy	DP	10	Dead
Johansson et al. [22]	1970	67	Female	1	Ocular	Head	NR	Biopsy	PD	11	Alive
Bianca et al. [23]	1991	48	Male	1	Unknown	Head	3	FNA	PD	12	Alive
Brodish et al. [24]	1993	75	Female	1	Cutaneous	Tail	5	CT	DP	12	Alive
Rütter et al. [9]	1994	55	Male	1	Unknown (1 year after surgery, melanoma detected in nasal cavity and nasopharynx)	Head	2.5	ERCP	DP	12	Alive
Sobesky et al. [25]	1997	32	Female	1	Thoracic melanoma	Diffuse infiltration	NR	ERCP, biopsy	No operation	1.5	Dead
Harrison et al. [26]	1997	NR	NR	1	NR	NR	NR	NR	NR	NR	NR
Medina-Franco et al. [27]	1999	60	Male	1	Unknown	Head	8	CT, US	PD	6	Dead
Wood et al. [19]	2001	NR	NR	8	NR	NR	NR	NR	Curative resection or palliative resection	Median 23.8	
		NR	NR	20	NR	NR	NR	NR	no operation	Median 15.2	
Hiotis et al. [28]	2002	NR	NR	1	NR	NR	NR	NK	PD	NR	Dead
Camp et al. [29]	2002	62	Female	1	Ocular	Body	5	CT, PET-CT	DP	20	Alive
Dewitt et al. [30]	2003	33	Male	2	Unknown	Head	5	EUS-FNA	Palliative gastrojejunostomy	6	Dead
		83	Female		Unknown	Tail	3	EUS-FNA	No operation	10	Alive
Mizushima et al. [31]	2003	51	Female	1	Cutaneous	Head	5	Biopsy	No operation	NR	NR
Nikfarjam et al. [32]	2003	45	Male	2	Ocular	Head	3	CT, MRI, PET-CT etc.	PD	6	Alive
		55	Male		Ocular	Head, body, tail	NR	CT, PET-CT etc.	TP	7	Alive
Carboni et al. [33]	2004	55	Female	1	Cutaneous	Head	8	Biopsy	PD	4	Dead
Crippa et al. [34]	2006	36	Female	1	NR	Head	NR	NR	PD	14	Dead
Belágyi et al. [35]	2006	28	Female	1	Ocular	Body	NR	CT	Pancreatic enucleation etc.	4	Dead
Eidt et al. [36]	2007	NR	NR	4	NR	NR	8	NR	PD	76	Alive
		NR	NR		NR	NR	5	NR	PD	30	Alive
		NR	NR		NR	NR	7	NR	PD	12	Dead
		NR	NR		NR	NR	5	NR	PD	25	Dead
Reddy et al. [37]	2008	NR	NR	3	NR	NR	Median size 4	NR	NR	Median 10.8	
Dumitraşcu et al. [7]	2008	43	Female	1	Ocular	Body	2	EUS-FNA	CP	12	Alive
Lanitis et al. [38]	2010	69	Male	1	Cutaneous	Head	4.5	CT	PD	96	Alive
He et al. [39]	2010	39	Male	1	Ocular	Tail	18	CT, MRI, ERCP etc.	DP	25	Alive
Vagefi et al. [8]	2010	57	Female	1	Ocular	Tail	2.2	EUS-FNA	DP	NR	NR
Portale et al. [4]	2011	43	Female	1	Unknown	Tail	1.7	US, CT, PET-CT	DP	NR	NR
Moszkowicz et al. [40]	2011	44	Female	1	Cutaneous	Uncinate process, Cephalo-isthmic junction	1.3, 0.9	Biopsy under EUS	PD	NR	NR
Sperti et al. [41]	2011	48	Male	1	Unknown	Body	2.9	CT	DP	24	Dead
Goyal et al. [18]	2012	47	Female	5	Cutaneous	Head	3	ERCP-assisted biopsy	PD	15	Dead
		73	Female		Cutaneous	Head	4	CT	PD	3	Dead
		58	Female		Unknown	Head	10	CT-guided biopsy	PD	11.4	Dead
		28	Female		Cutaneous	Head	2	PET-CT	PD	4.5	Dead
		69	Male		Unknown	Tail	4.5	Biopsy	DP	26	Dead
Larsen et al. [2]	2013	32	Female	1	Cutaneous	Head	NR	CT	PD	228	Alive
Birnbaum et al. [5]	2013	45	Female	1	Cutaneous	Head	6	Biopsy	PD	19	Alive
Sugimoto et al. [10]	2013	46	Male	1	Nasal cavity	Body	3.3	CT, PET-CT	DP	10	Dead
Solmaz et al. [42]	2014	59	Male	1	Cutaneous	Head	3.8	Biopsy	No operation	NR	NR
Jana et al. [1]	2015	75	Male	1	Cutaneous	Head, body	2.4, 1.4, 1, 0.6	EUS-FNA	No operation	NR	NR
De Moura et al. [3]	2016	58	Female	1	Ocular	Head, neck	3.1	EUS-FNA	PD	NR	NR
Nadal et al. [43]	2016	57	Female	1	Ocular	Tail	2	EUS-FNA	NR	NR	NR
Ben Slama et al. [44]	2017	55	Female	1	Unknown	Head	5.5	CT, MRI	PD	15	Alive
Liu et al. [45]	2018	54	Male	1	Cutaneous	Head	3.1	CT	PD	6	Alive
Current	2019	67	Female	1	Nasal cavity	Body	3.5	EUS-FNA	DP	24	Alive

*NR* not reported, *PD* pancreatoduodenectomy, *DP* distal pancreatectomy, *TP* total pancreatectomy, *CP* central pancreatectomy

Despite technological advances, preoperative diagnosis of metastatic pancreatic tumor is sometimes difficult [11]. Metastatic lesions from malignant melanoma have hypervascularity on contrast-enhanced CT and MRI [12]. The blood supply to metastatic lesions is carried from the surrounding organs; therefore, the surrounding tissue of the large lesion receives more blood supply than the central area, resulting in rim enhancement, especially in lesions larger than 1.5 cm. The same could be said in our case, as a high-attenuation rim was revealed on enhanced CT. EUS provided us with high-quality images to examine the pancreas and nearby structures. In general, pancreatic metastases on EUS have regular margins and appear as homogenous structures that are hypoechoic compared with the surrounding pancreas [13]. In our case, EUS revealed the mass to be hypoechoic and homogenous with the central anechoic areas. Few studies have reported on CE-EUS findings of pancreatic metastatic lesion. Pancreatic metastasis of renal cell carcinoma tends to show hyperenhancement, whereas malignant melanoma may or may not show hyperenhancement [13–15]. The lack of characteristic findings makes diagnosis of metastatic malignant melanoma by CE-EUS difficult.

To confirm the diagnosis of pancreatic metastatic lesions, pathological examination is necessary. EUS-FNA plays an important role in providing cytological/histological diagnosis, and it is extremely useful in identifying pancreatic metastases. To distinguish pancreatic metastases from a primary carcinoma accurately, effective sampling and immunocytochemistry are needed [1, 3, 6]. EUS-FNA with rapid on-site evaluation provides effective sampling, because a cytopathologist can ensure that the samples are adequate for assessment [16]. Immunohistochemical analysis has been shown to be useful in identifying metastatic melanoma; the sensitivity of S100, Melan A, and HMB-45 are reported to be 97–100%, 75–92%, and 69–93%, respectively. The specificity of S100 and Melan A is reported to be 75–87% and 95–100%, respectively [17]. In our case, Melan A and HMB-45 were positive.

The prognosis of patients with malignant melanoma metastatic to the pancreas is unknown, although metastatic melanoma usually indicates poor prognosis [5]. There are few experiences with pancreatic resection for isolated pancreatic metastases, and pancreatic resection is controversial. Some studies have shown that complete surgical resection of a localized metastatic disease can prolong survival [5, 18]. However, Wood et al. [19] reported 28 patients with isolated pancreatic metastases from malignant melanoma and found that the 5-year survival rate of pancreatic resection (performed in 8 patients) was 37.5% (median survival, 23.8 months), as compared with 23% (median survival, 15.2 months) of the 20 patients treated with non-resection. It is critical that surgery should be performed only when a complete resection is possible. Therefore, exhaustive preoperative staging is needed to confirm both the absence of local invasion of the major vasculature and the absence of distant metastasis. PET scan has a high sensitivity and specificity for detecting metastasis from malignant melanoma [20]. In our case, PET-CT also had an important role; preoperative PET-CT identified the pancreatic tail mass, and the 6-month postoperative PET-CT showed high uptake in the left nasal cavity and peritoneum.

In conclusion, this unique case of isolated pancreatic metastasis from malignant melanoma was conclusively proven with EUS-FNA prior to the diagnosis of the primary lesion. Broad differential diagnoses should be considered when faced with inconclusive imaging studies of pancreatic tumors. In such cases, EUS-FNA is useful in providing a definitive diagnosis.
